# Optimal Scaling Approaches for Perfusion MRI with Distorted Arterial Input Function (AIF) in Patients with Ischemic Stroke

**DOI:** 10.3390/brainsci12010077

**Published:** 2022-01-05

**Authors:** Sukhdeep Singh Bal, Fan Pei Gloria Yang, Yueh-Feng Sung, Ke Chen, Jiu-Haw Yin, Giia-Sheun Peng

**Affiliations:** 1 Department of Mathematical Sciences, University of Liverpool, Liverpool L69 3BX, UK; Sukhdeep.singh.bal@liverpool.ac.uk (S.S.B.); K.Chen@liverpool.ac.uk (K.C.); 2 Center for Cognition and Mind Sciences, National Tsing Hua University, Hsinchu 300, Taiwan; 3 International Intercollegiate Ph.D. Programme, National Tsing Hua University, Hsinchu 300, Taiwan; 4 Department of Foreign Languages and Literature, National Tsing Hua University, Hsinchu 300, Taiwan; 5 Department of Radiology, Graduate School of Dentistry, Osaka University, Osaka 565-0871, Japan; 6 Department of Neurology, Tri-Service General Hospital, National Defense Medical Center, Taipei 114, Taiwan; sungyf@ndmctsgh.edu.tw (Y.-F.S.); ch9135@gmail.com (J.-H.Y.); tsghpeng@gmail.com (G.-S.P.); 7 Department of Neurology, Taipei Veterans General Hospital, Hsinchu Branch, Hsinchu 310, Taiwan

**Keywords:** PVE (partial volume effect), CBF (cerebral blood flow), AIF (arterial input function), CBV (cerebral blood volume), SVD (singular value decomposition)

## Abstract

Background: Diagnosis and timely treatment of ischemic stroke depends on the fast and accurate quantification of perfusion parameters. Arterial input function (AIF) describes contrast agent concentration over time as it enters the brain through the brain feeding artery. AIF is the central quantity required to estimate perfusion parameters. Inaccurate and distorted AIF, due to partial volume effects (PVE), would lead to inaccurate quantification of perfusion parameters. Methods: Fifteen patients suffering from stroke underwent perfusion MRI imaging at the Tri-Service General Hospital, Taipei. Various degrees of the PVE were induced on the AIF and subsequently corrected using rescaling methods. Results: Rescaled AIFs match the exact reference AIF curve either at peak height or at tail. Inaccurate estimation of CBF values estimated from non-rescaled AIFs increase with increasing PVE. Rescaling of the AIF using all three approaches resulted in reduced deviation of CBF values from the reference CBF values. In most cases, CBF map generated by rescaled AIF approaches show increased CBF and Tmax values on the slices in the left and right hemispheres. Conclusion: Rescaling AIF by VOF approach seems to be a robust and adaptable approach for correction of the PVE-affected multivoxel AIF. Utilizing an AIF scaling approach leads to more reasonable absolute perfusion parameter values, represented by the increased mean CBF/Tmax values and CBF/Tmax images.

## 1. Introduction

Dynamic susceptibility contrast (DSC) MR perfusion imaging refers to perfusion scanning after an intravenous injection of a paramagnetic contrast agent containing gadolinium [1,2,3,4]. By utilizing the signal loss during the passage of a contrast agent through the brain tissues, it is possible to estimate perfusion parameters that are essential to identify ischemic core and penumbra, such as the cerebral blood flow (CBF), cerebral blood volume (CBV), mean transit time (MTT) and perfusion–diffusion mismatch [2,3,5,6]. Diagnosis and timely treatment of ischemic stroke increasingly rely on the fast and accurate quantification of these parameters as there is a short time window for the diagnosis as well as the administration of treatment therapies [6,7]. DSC perfusion imaging has proven successful in a variety of clinical studies on diagnosis of ischemic stroke and reperfusion [7,8,9]. Despite the large amount of research, inaccurate quantification still remains a challenge that may lead to erroneous diagnosis due to problems associated with image postprocessing and quantification approaches.

In perfusion quantification, one of the most important functions, which is required as input from the perfusion data, is the arterial input function (AIF) [10]. AIF is the function that describes contrast agent concentration over time as it enters the brain through the brain feeding artery [1,2]. When the voxel or region that has been selected for quantification of AIF has only some fraction of the arterial blood, the partial volume effect (PVE) arises. During measurement of AIF, spatial resolution used in perfusion MRI and the average size of major arteries make a degree of partial volume unavoidable [3,10,11]. The arterial and tissue contributions are complex numbers with amplitude and phase. The magnitude and phase of tissue components in the selected AIF voxel may decide whether there will either destructive or constructive contributions by the tissue components [10].

**Figure 1:**
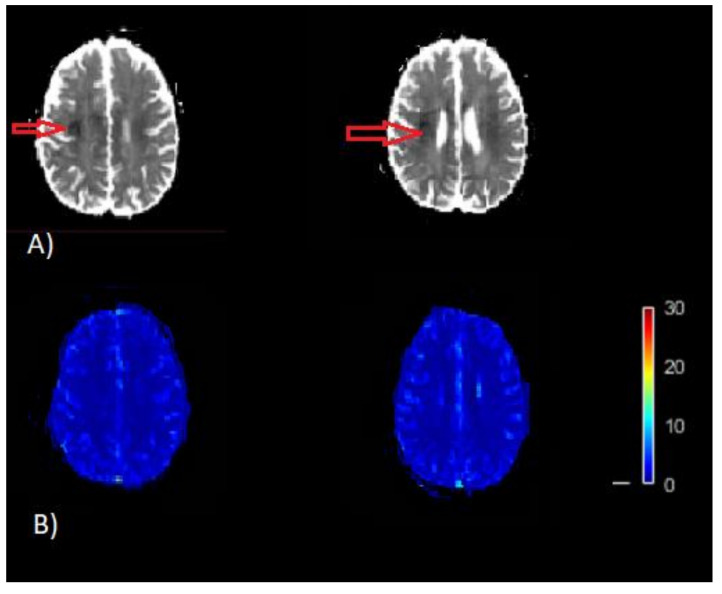
Example of misleading perfusion parameter maps. (**A**) Apparent diffusion coefficient (ADC) image (${mm}^{2}$/s). The dark region on ADC image thresholded by ADC ≤ 620 × 10^−6^ ${mm}^{2}$/s is the infracted core (red arrow) on the map. (**B**) CBF map (bottom) [mL/100 g/min]. This CBF map does not indicates the infract region as represented on the ADC map, which is a result of inaccurate quantification of CBF.

The perfusion model uses AIF as an initial input to calculate perfusion parameters as final output [10]. In the process of calculation of perfusion imaging parameters, the PVE seriously affects the estimation of arterial input function (AIF) [11,12,13,14,15,16]. As voxels with signals from both artery and surrounding tissues may result in distortion of the signal loss of the contrastive agent during the passage of blood, this may lead to erroneous estimation of AIF, which consequently yields misleading brain maps of CBF and Tmax. In current clinical practices, volumes on brain image with Tmax greater than 6 s are considered to be the critically hypoperfused region, which is also known as the penumbra, and tissues with relative CBF <30% are considered to be the infarct core [17]. Misleading CBF and Tmax brain images may fail to identify infract regions as well as hypoperfused regions (Figure 1). Early and correct assessment of the hypoperfused as well as infract regions are critical for appropriate diagnosis and treatment decisions in acute stroke [11,16,17].

In light of the substantial influence of the PVE on AIF as well as perfusion parameters, the present study investigates potential approaches for minimization of the volume-averaging artifacts associated with the PVE. We hypothesize that correct estimation and reasonable perfusion parameters can be achieved by several rescaling methods of AIF. This enhancement could be evidently seen from the Tmax and CBF images before and after rescaling.

This proposed enhancement method will use multiplicative rescaling on the multiple AIF voxels to minimize the underestimation or overestimation of AIF and CBF values. By increasing the size of the region from which the AIF is sampled, we will demonstrate the increase in the PVE and the increased distortion of AIF estimation. Three different multiplicative rescaling approaches are used to rescale the AIF, as follows: (a) scaling using AIF curve; (b) scaling using VOF curve; (c) scaling by matching peaks. The rescaling factor is decided according to different rescaling approach and is applied to the AIF concentration curves. Finally, the variation in CBF value estimated from the reference AIF with minimal PVE and the AIF concentration curve after rescaling is evaluated. Based on these comparisons, an optimal scaling method to minimize the PVE is determined and the perfusion parameter maps are generated. It is anticipated that the scaling approach will generate rational parameters, as it takes into factor the conservation of the time integral of the tracer concentration curve, C(t), through the vasculature, which might affect the AIF calculation most.

## 2. Materials and Methods

### 2.1. Data Acquisition

Fifteen patients suffering from acute ischemic stroke underwent perfusion imaging as a part of their diagnostic MR procedure. A single-shot gradient–echo EPI sequence (TR/TE/flip angle/number of slices/voxel size: 1800 ms/40 ms/60°/23/1 × 1 × 5 mm) on a clinical 1.5 T MR scanner (Signa; General Electric) was used to acquire contrast-enhanced T2*-weighted images from the Tri-Service General Hospital, Taipei. During perfusion imaging, with the speed of 5 mL $s^{-1}$, a dose of 20 mL of bolus injection (Magnevist; gadopentetate dimeglumine, Bayer Health Care pharmaceuticals Inc, Berlin, Germany.) was injected. The present study was granted the IRB approval by the Tri-Service General Hospital, Taipei, Taiwan.

### 2.2. Approaches for Correction of the Artifacts That Arise from the PVE

Voxels containing signal contributions from both the artery and the surrounding tissues are referred as voxels affected by the PVE [12,13]. Since the signals come from the artery and the neighboring tissues, we first define the signals from these regions. Suppose we have PVE-affected voxels, which are selected for AIF estimation; they are composed of k and t fractions of arterial blood signal ($S_{a})$ and tissue signal $\left(S_{t}\right)$. The MR signal from the entire voxel ($S_{v}$) then reflects the weighted average of signals $S_{a}$ and $S_{t}$, as follows:

(1) $${\;S}_{v}=\;k\;S_{a}+t\;S_{t}$$

It may be the case that the head of the person was not static throughout the whole acquisition. In this case, the effect of the PVE may be associated with the pose of the head, with respect to the sampling grid, and ‘k’ in Equation (1) might be potentially time dependent [18,19]. PWI contrast DICOM images were co-registered to a common template for all subjects after the acquisition. If it is assumed that the tissue contribution is much smaller than the arterial contribution (k$S_{a}\gg {\mathrm{tS}}_{t}$), then multiplicative rescaling can be used to estimate correct arterial signal from the measured voxel signal by multiplication with the inverse volume fraction of arterial blood [13] i.e.,

(2) $${\;S}_{a}=S_{v}/k$$

A direct evaluation of Equation (2) is not possible since k is unknown. The present study implements three different criteria to determine the rescaling factor, i.e., k of Equation (2). The MR signals are converted to concentration curves based on a traditional nonlinear relationship provided by earlier studies [2,10].

(3) $$S(t)=S_{0}e^{-\frac{\mathrm{TE}}{p}C\left(t\right)}$$

$S_{0}$ is the baseline (pre-bolus or pre-contrast agent) MR signal intensity, TE is the echo time, p is the proportionality constant taken as p = 1 [4], and C(t) represents the concentration time function. A direct expression for concentration values based on MRI signal data is derived by inverting Equation (3), as follows:

(4) $$C(t)=-(p/\mathrm{TE})\;\ln\;(S(t)/S_{0})$$

A reference arterial curve derived from a voxel with minimal PVE is manually selected. The MR concentration from the entire voxel ($C_{v}$) reflects the weighted average of concentration in artery $\left(C_{a}\right)$ and concentration in tissue ($C_{t})$, which can be expressed as C_v_ = k C_a_ + t C_t_. Assuming that the concentration in tissue is much smaller than the concentration in the artery, this can be simplified as C_v_ = k ∗ C_a_. Following this argument, k is estimated from concentration values, as follows:

(5) $$\int_{0}^{\infty }C_{\mathrm{ref}}\;(t)\;\mathrm{dt}=\int_{0}^{\infty }C_{a}\;(t)\;\mathrm{dt}=\int_{0}^{\infty }C_{v}\;(t)/k\;\mathrm{dt}$$

The first two parts of the equation imply that AIF concentration time curve ($C_{a}$) has the same area under the curve (AUC) as any other manually selected reference concentration time curve ($C_{\mathrm{ref}}$) [20]. The relationship of conversion of MRI signal to concentration values is nonlinear (Equations (3) and (4)). In this study, we intend to make use of AUC of concentration curves rather than the signal curves to derive scaling factor ‘k’. So, we assume that during the calculation of AUC of arterial curve the non-linearity of signal to concentration conversion will have minimal effect. On the similar pattern of Equation (2), AUC of arterial curve would be ratio of AUC of the concentration curve from multiple voxels to the scaling factor, this is represented analytically in last two parts of Equation (5).

The first rescaling approach referred as scaling by AIF uses concentration curve of a reference AIF as ($C_{\mathrm{ref}}$) and concentration curve of selected multivoxel AIF region as ($C_{v}$) in Equation (5) to estimate the scaling factor k. The second rescaling approach referred as scaling by VOF uses concentration curve of a venous output function (VOF) as $C_{\mathrm{ref}}$ and concentration curve of selected multivoxel AIF region as ($C_{v}$) in Equation (5) to estimate the scaling factor k. The first two rescaling approaches are based on the principle of conservation of time integral of tracer concentration curve C(t) through the vasculature. The third rescaling approach, referred to as scaling by peak, estimates the scaling factor k by matching the peak height of the multi-voxel AIF concentration time curves with the reference concentration time curve. This follows that the multivoxel AIF will have similar characteristics to any other concentration curve in terms of peak height [13].

### 2.3. Reference AIF Curve

**Figure 2:**
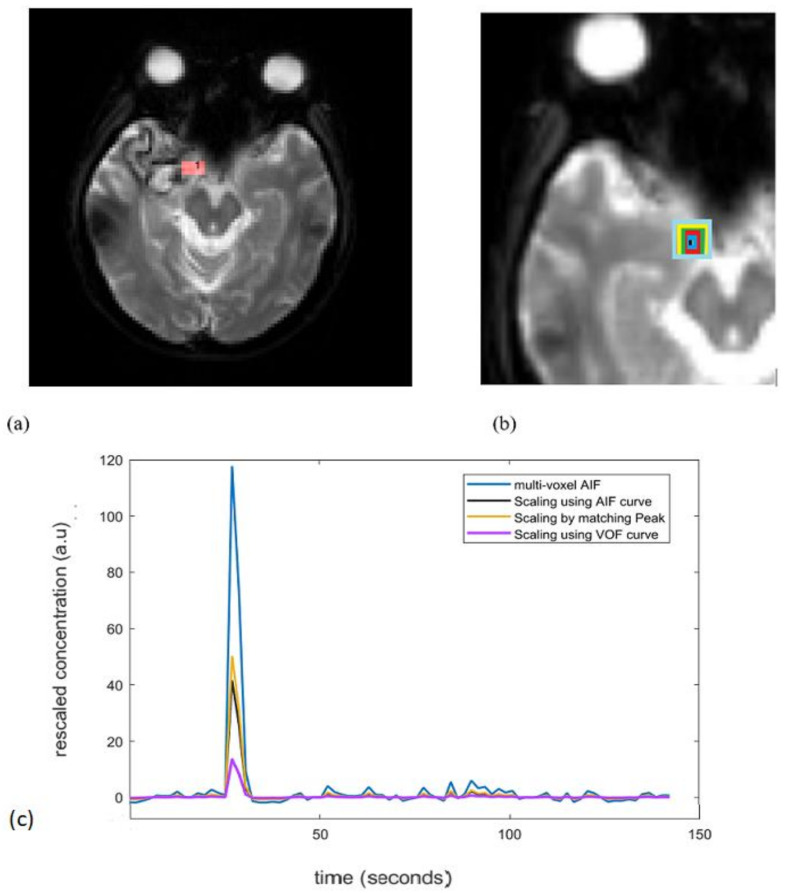
(**a**) Red colored square represents ICA used for reference AIF determination. (**b**) Increased size of the AIF, i.e., AIF concentrations were measured from 3, 5, 7, 9, and 11 voxels centered around the reference ICA voxel shown by black, blue, red, green, yellow, light blue colored squares, respectively. (**c**) An example of non-corrected AIFs (3 voxel AIF; blue curve) and corrected AIFs by all 3 scaling approaches. For a single subject, the unscaled AIF was derived from a 3-voxel-wide region to include the effect of the PVE. ICA: internal carotid artery; PVE: partial volume effect.

During PWI-MRI, the internal carotid artery (ICA) is nearly perpendicular to the axial plane and offers the advantage of easy and reliable manual identification with minimal errors from volume-averaging artifacts [10]. As demonstrated in Figure 2a, internal carotid artery (ICA) is used as $C_{\mathrm{ref}}\;$ in the model Equation (5), as it is associated with minimal errors from volume-averaging artifacts. The increased size of the AIF sampling region represents the increased degree of the PVE. The AIF concentration was measured from 3, 5, 7, 9, and 11 voxels centered around the reference ICA voxel (Figure 2b).

### 2.4. Perfusion Analysis

The reference AIF curve ($C_{\mathrm{ref}}$) for the rescaling approach, was measured from an ICA voxel where the concentration curve had the features of large amplitude (peak), small width, and fast attenuation. The venous output function (VOF) is the concentration–time curve measured in a vein that drains the organ of interest. Based on practical and theoretical considerations, manual VOF is often chosen from the sagittal or transverse sinus [10,13,17]. To obtain a VOF with peak from the first-pass bolus passage followed by a recirculation peak, voxel with the maximum signal in the sagittal sinus is chosen as the reference VOF curve. To reproduce AIFs with an increasing degree of the PVE, we used concentration curves measured from ROIs of widths 3, 5, 7, 9, and 11 voxels, centered on the reference AIF voxel for 15 patients (Figure 2b). A region of interest (ROI) tissue was manually selected in the grey matter [21] to evaluate the CBF percentage change (∆CBF (%)). ∆CBF is the percentage change of CBF estimated from rescaled AIFs relative to the CBF estimated from reference AIF curve. When using the VOF approach, the reference AIF concentration curve (width of 1 voxel) was also rescaled with the reference VOF curve.

The area under the curve estimation, as well as the perfusion quantification for CBF, was performed by deconvolution of the tracer kinetic equation (Equation (6)) [2,3,22], implemented using MATLAB scripts (MathWorks, Natick, MA, USA).

(6) $$C_{t}\left(t\right)\;=\;\mathrm{CBF}(C_{a}\left(t\right)\;⊗\;R\;(t))$$

(7) $$\mathrm{CBF}\;=\;\frac{1-H_{\mathrm{sv}}}{1-H_{\mathrm{lv}}}\;\frac{1}{ϼ}\;\max\left(R\left(t\right)\right)\;[\mathrm{mL}/100\;g/\min]$$

where $C_{t}\left(t\right)$ denotes the tissue concentration curve of the ROI located in gray matter, $C_{a}\left(t\right)$ is the AIF either corrected using one of the three rescaling criteria described above or without rescaling, symbol ⊗ represents the convolution operator, and R (t) represents the residue impulse response function. Deconvolution of Equation (6) to estimate CBF was carried out using the block circulant singular value decomposition method (cSVD) [2,22,23]. The block circulant decomposition method has an advantage of being less sensitive to tracer arrival timing differences. Deconvolution of Equation (6) for known values of $C_{a}\left(t\right),C_{t}\left(t\right)$ leads to evaluation of the residue function R(t). CBF is measured as the maximum of R(t). Furthermore, in Equation (7), $H_{\mathrm{sv}}$ and $H_{\mathrm{sv}}$ represent a correction for different levels of hematocrit in large vessels and small vessels. Here, the values used are $H_{\mathrm{sv}}$ = 0.25, $H_{\mathrm{lv}}$ = 0.45 and ϼ = 1.04 g/mL (density of the brain) [21,24]. Tmax is the time, ‘t’, for which R(t) attains maximum value. After estimating CBF and Tmax for all brain tissues, CBF and Tmax are represented visually on axial brain maps.

## 3. Results

Figure 2c is an example to see the effect of the AIF correction by all the three scaling approaches. For a single subject, the unscaled AIF was derived from a 3-voxel-wide region to include the effect of the PVE. After correction, the peak of unscaled multi voxel AIF (3 voxels wide) reduces for all the approaches and there is slight change in the tail (recirculation) part. The VOF approach rescales the AIF to large extent by reducing the overestimated peak.

**Figure 3:**
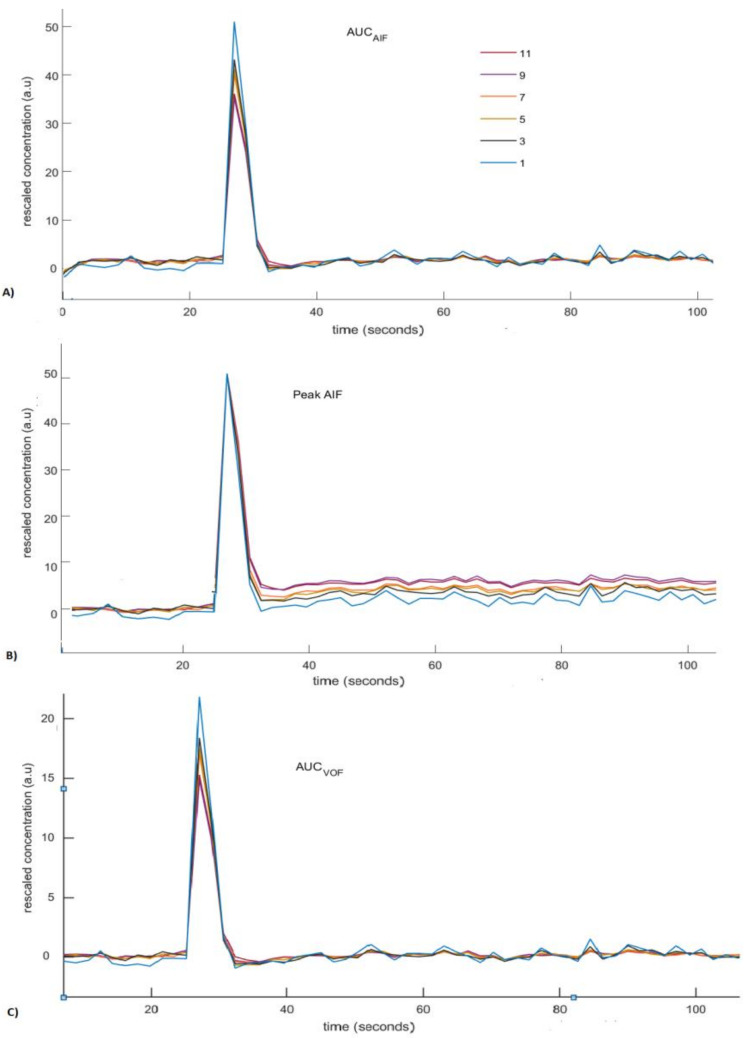
Rescaled concentration curves of arterial input functions (AIFs) generated using different scaling methods. The legend in (**A**) indicates the width (in voxels) of ROI used for measuring the AIF. (**A**) Rescaled AIFs generated using scaling by AIF approach. (**B**) Rescaled AIFs generated using scaling by matching peak height approach. (**C**) Rescaled AIFs generated using scaling by VOF approach.

This Figure 3 shows curves of rescaled AIFs with increasing degree of the PVE plotted as function of time using different scaling approaches. Overall, the deviation of the rescaled AIFs from the reference AIF increases with increasing PVE (i.e., increased number of voxels used for measuring AIF). Rescaling of AIFs by using scaling by AIF approach leads to small deviations at the tail, but large deviations at the peak (Figure 3A). Rescaling of AIFs by matching peak reproduces peak similar to reference AIF for rescaled AIFs. However, this approach fails to accurately reproduce the tail similar to the tail of reference AIF (Figure 3B). Rescaling of measured AIFs using scaling by VOF approach gives rise to results similar to the first approach apart from the decrease in the overall peak height estimates of rescaled AIFs (Figure 3C). The tail accounts for the recirculation of tracer in the brain vasculature after an initial bolus passage, whereas the peak represents the maximum amplitude bolus rush through the brain vasculature [10]. The ideal AIF concentration curve has to represent correct tail and peak in order to reproduce more reasonable perfusion parameter maps.

Rescaled AIFs curves do not coincide with the exact reference AIF curve (Figure 3). Rescaled AIFs match the exact reference AIF curve either at peak height or at tail. The least percentage change of CBF values estimated using rescaled AIFs from CBF values estimated using reference AIF may decide the most appropriate scaling approach. The least percentage change of CBF indicates the approach that will be least affected by the volume-averaging artifacts.

**Figure 4:**
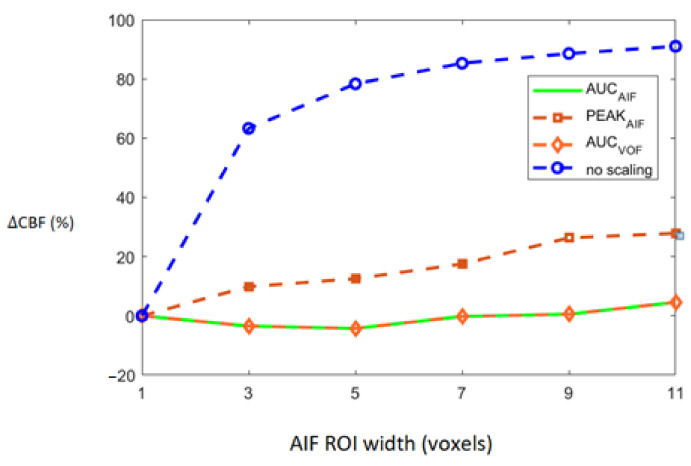
Average CBF divergence from reference CBF plotted against increasing number of voxels. Average CBF divergence for group of 15 patients is plotted according to increased partial volume effect (PVE) for all four scaling approaches indicated by the legend on right.

Deviation of CBF, based on all 15 patients, which is represented as ∆CBF, was estimated as percentage difference in CBF estimated using rescaled AIFs and reference AIF. Rescaling of the AIFs was conducted using the following four approaches: no scaling, scaling by AIF, scaling by VOF, and scaling by peak height. The increasing degree of the PVE and its association with ∆CBF in 15 patients is shown in Figure 4. As shown in the figure, ∆CBF values estimated from non-rescaled AIFs without any modification increases as the number of voxels used for measuring AIF increases. This shows that the increased PVE resulting from the increased number of voxels might seriously affect the estimation of AIF and consequently the calculation of perfusion parameters. Rescaling of the AIF using either of the three approach results in the reduced ∆CBF (%) values. Overall, scaling by AIF and scaling by VOF seemed to achieve the best and similar results as they yield the least ∆CBF (%) values when the PVE increases maximally among all scaling approaches.

**Figure 5:**
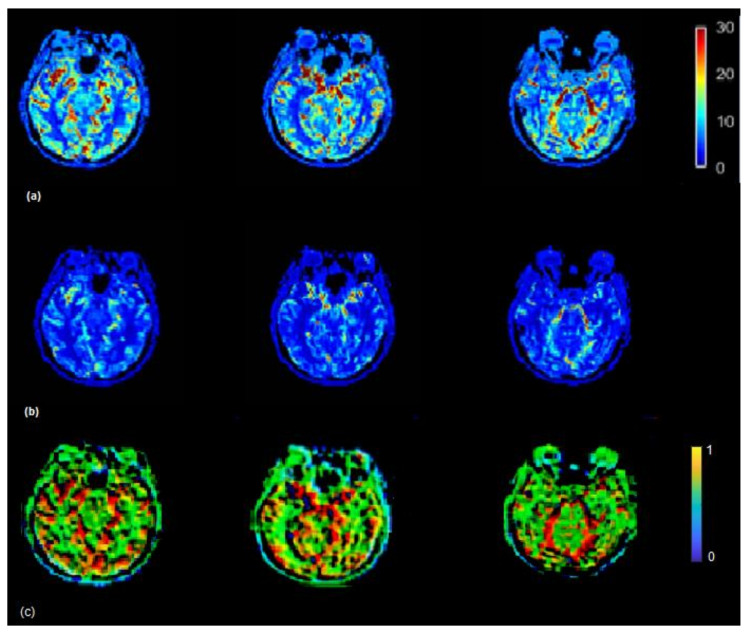
(**a**) CBF (mL/100 g/min) map generated by using rescaled AIF (**b**) and non-rescaled AIF (bottom). CBF map generated using rescaled AIF represents increased CBF values in the shown axial brain slices. CBF maps from non-rescaled AIF display mostly all the ROIs with decreased blood flow which makes it difficult to locate the regions which actually have a decreased flow. CBF images derived using rescaled AIF display ROIs with increased flow (red color) which helps to segregate the regions with decreased blood flow. This may help clinicals to identify the infract regions as well as regions with decreased blood flow on visual brain CBF images. (**c**) Maps illustrating the ratio between CBF values derived from the scaled and the non-scaled AIF.

The CBF brain map was generated in the absence of scaling of AIF as well as by using the VOF rescaling approaches (Figure 5). In some cases, relative to the CBF map generated by using non-rescaled AIF, the CBF map generated by rescaled AIF approaches showed increased CBF values on the slices in the left and right hemispheres (red color) (visible in Figure 5). From Equations (5) and (6), it follows that the ratio of scaled and unscaled CBF values should be the scaling factor k. The images of the ratio of scaled and unscaled maps are expected to show the factor k for every voxel (Figure 5c). The mean CBF values, based on all 15 subjects using non-rescaled (AIF ROI width = 5 voxels) and VOF approach, were 43.98 and 61.16 mL/100g/min, respectively. The mean CBF values for AIF-rescaled and peak scaled approach were 57.10 and 47.10 mL/100g/min, respectively. At individual level, all the fifteen patients in this study did not follow similar pattern of underestimated CBF values due to the PVE. This has been demonstrated by studying the association of increasing degree of the PVE with deviation of CBF (Supplementary Material, Figure S1). To generalize, we need a larger dataset to conclude whether the scaling corrects the underestimation of CBF, as this could vary patient to patient in a small cohort.

**Figure 6:**
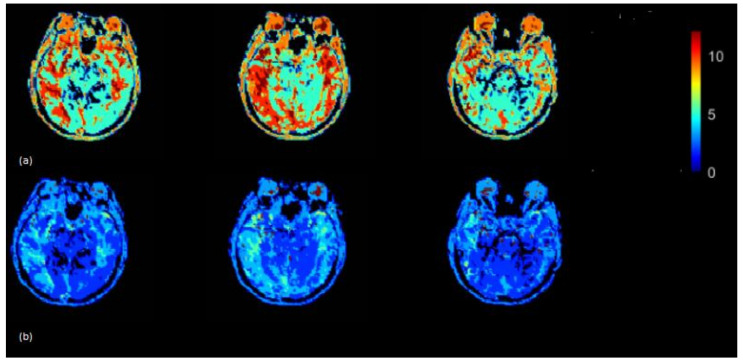
Tmax (seconds) map generated by using rescaled AIF (**a**) and non-rescaled AIF (**b**) for one subject. Tmax map generated using rescaled AIF represents increased values in the shown axial brain slices.

The Tmax (seconds) map was generated using the rescaled AIF (VOF approach) (Figure 6) (top) and non-rescaled AIF (Figure 6) (bottom). The Tmax map generated using the rescaled AIF showed increased values in the axial brain slices in the left and right hemispheres. The mean Tmax values (range of 0–12 s) based on 15 subjects using non-rescaled (AIF ROI width = 5 voxels) and VOF approach were 4 s and 7 s, respectively. The derivation of Tmax is performed from the residue function (R(t)), which is achieved by deconvolution of Equation (6). Tmax is the argument, i.e., ‘t’ of the maximum of R(t). The deconvolution utilizes a matrix method called the circulant singular deconvolution, which is sensitive to the peak amplitudes of AIF (cSVD) [23]. The changed AIF amplitude used in the cSVD algorithm shifts the maximum of R(t) to a higher time points ‘t’ which accounts for higher Tmax values. The change in Tmax is consistent with a previous study where different AIFs with changed amplitudes and similar shape selected by different algorithms resulted in change of Tmax values [1]. The increased Tmax maps generated by the rescaled AIF may allow clinicals to visualize the critically hypoperfused regions which are likely to be salvageable.

## 4. Discussion

In the present study, we used multiple AIF rescaling approaches using perfusion imaging data so as correct the amplitude falsification of the multi-voxel AIF. This, thorough investigation, has allowed us to study the effect of the PVE on a multi-voxel AIF, which is a prerequisite for obtaining accurate CBF measurements using MR bolus tracking [12]. The significant findings of the study revealed that rescaling AIF using scaling by VOF approach leads to more reasonable absolute perfusion parameter values, represented by the increased mean CBF/Tmax values and CBF/Tmax images. This may assure that the core or brain regions with decreased blood flow will not be overlooked. The present study has shown that the absence of multi-voxel AIF scaling results in inaccurate and untrue CBF values.

The spatial resolution typically used in the cerebral DSC-PWI, 1.9 × 1.9 × 5 mm^3^ makes it difficult to identify vessels and only voxels placed in the ICA could be selected as reference AIF, free from partial volume of the tissue [25]. Selecting more voxels or a large region for AIF estimation can lead to significant PVE. However, PVE-corrected, multi-voxel AIF is necessary as AIF obtained from a single voxel or a small region is not reliable enough due to noise in spatial measurements and motion in temporal measurements [1]. In this study, we intend to make use of scaling as a way out to calibrate a multiple voxel AIF which would further lead to reduce the effect of the PVE on the quantification of absolute CBF and Tmax values. The great benefit associated with this type of linear scaling is that predefined thresholds could be used for evaluation/comparison of perfusion images and parameter values obtained from different scanners examined at different time points.

For the typical spatial resolution used in DSC-MRI studies, average size of major arteries and considering that the true AIF as signal is saturated at peak concentrations for a voxel with 100% blood, a degree of partial volume is in practice unavoidable when measuring the AIF [10]. The signals from the arterial and tissue contributions are complex numbers (with amplitude and phase), which makes selection of PVE-free AIF more complicated [10]. The scaling method used in this study might be a pragmatic way of using a multi-voxel AIF. Considering the difficulty involved in the selection of PVE-free reference AIF, we can consider that reference AIF in the study is an approximation of the true AIF with minimal PVE.

Clinically, AIF selection depends on the expertise, experience, and skill of experts accompanied by time consumption, low reproducibility, and often including tissue signals in AIF. In the past, perfusion studies utilized AIF selection approaches such as slice-specific AIF selection, clustering methods that require ROI to be marked manually prior to AIF extraction [26,27,28,29], and multi stream 3D CNN method [1,30]. No matter what selection strategy is taken, the PVE is always present as the voxels selected may exhibit partial signals. Therefore, a proper approach must be taken to solve the problem. Rescaling of AIF discussed in the present study can be carried out for PVE correction even if the AIF selection procedure is slice specific.

The extent of the influence of PVE on the output perfusion parameters has been observed in previous research. In vivo studies have reported large variations in perfusion parameters due to the PVE [12,13,25,31]. Past simulation results in DSC MRI proved that uncorrected AIF measured with a partial volume fraction of about 50% could produce a four times CBF overestimation along with distortions of AIF frequency characteristics [12]. Investigation of the impacts of the PVE on quantitative perfusion metrics at 1.5 T and 3.0 T has reported broaden tissue contribution, resulting in fluctuations in the AIF which further compromises quantitative perfusion estimates in a nonlinear fashion [25].

Inaccurate AIF estimation can be minimized by correction for partial volume effects by utilizing specific post processing approaches or data acquisition techniques. Past MRI research has reported that correction for the PVE was appropriate for arteries that were parallel to the main magnetic field by estimation and subtraction of the static signal of the surrounding tissue [31]. However, to measure quantitative input function of vessels that were not parallel to the main magnetic field was still a challenge [31]. CTP studies suggested that AIF measurements should be done with smaller section thickness, i.e., small location or reduced voxel volume, as AIF and VOF measurements from thicker sections would cause an overestimation of CBV and CBF [32,33]. In vivo MRI studies have suggested that minimal impact of the PVE in AIF measurements may be achieved with reduced contrast dosage and minor adjustments to the pulse sequence [12]. Previous research used linear scaling methods for the correction of PVE-affected AIF on T1-weighted perfusion imaging datasets as they allow easy identification of the large arteries [13]. Rescaling using VOF as a reference was reported as most feasible approach [13]. The present study used T2-weighted perfusion imaging datasets and used rescaling on AIF concentration curves rather than the MR signal curves and proved to be consistent with past results of PVE correction of T1-weighted datasets.

Recent studies in DCE-MRI demonstrate that inter-frame realignment have a huge effect on parameter mapping [34]. In this study, reference AIF (red colored square represented on ICA (Figure 2a)) and ROI used as multivoxel AIF (i.e., AIF concentrations measured from 3, 5, 7, 9, and 11 voxels centered around the reference ICA voxel shown by black, blue, red, green, yellow, light blue squares, respectively, in Figure 2b) were measured on a same brain MRI axial slice for all subjects. For the geometrical alignment of axial brain slice selected for AIF determination, co-registration to a common template for all subjects after the acquisition was considered. However, in case if multiple axial slices are utilized for AIF selection across the subjects, then inter-frame realignment has to be considered due to its impact on parameters. The limitation of the present study is that multiplicative rescaling can only be used if tissue contribution is much smaller than the arterial contribution. This condition restricts AIF correction in case when there is ample amount of surrounding signal contributions to the AIF. The use of scaling factor assumes that any PVE in the AIF can be represented by linear scaling; however, in the case of large signal contribution from surrounding tissue, the PVE can be very complex and can distort the AIF shape.

## 5. Conclusions

In summary, the present study demonstrates that utilizing scaling approach provides more reasonable absolute perfusion parameter values, represented by the increased mean CBF/Tmax values and CBF/Tmax images. This will assure that the core, as well as the infract region, will not be overlooked. Distortions due to the PVE might be still present in AIF after the scaling as it does not affect the shape of the curve to a large extent. Rescaling AIF by VOF approach seems to be the robust and adaptable approach for correction of the PVE-affected multivoxel AIF. Absence of multi-voxel AIF scaling during deconvolution of the tracer kinetic equation may lead to inaccurate CBF values.

## Data Availability

The data that support the findings of this study are available from the corresponding author upon reasonable request.

## Supplementary Materials

The following supporting information can be downloaded at https://www.mdpi.com/article/10.3390/brainsci12010077/s1, Figure S1: The association of increasing degree of PVE with deviation of CBF for three patients.
