# Supplementary material for: Optimal Scaling Approaches for Perfusion MRI with Distorted Arterial Input Function (AIF) in Patients with Ischemic Stroke
Source: Brain Sci. 2022 Jan 5;12(1):77. doi: 10.3390/brainsci12010077 (PMC8774085; doi:10.3390/brainsci12010077)
Supplement: Supplementary file 1 [file brainsci-12-00077-s001.zip › brainsci-1467315-supplementary.pdf]

Supplementary Material :

Association of increasing degree of PVE with deviation of CBF

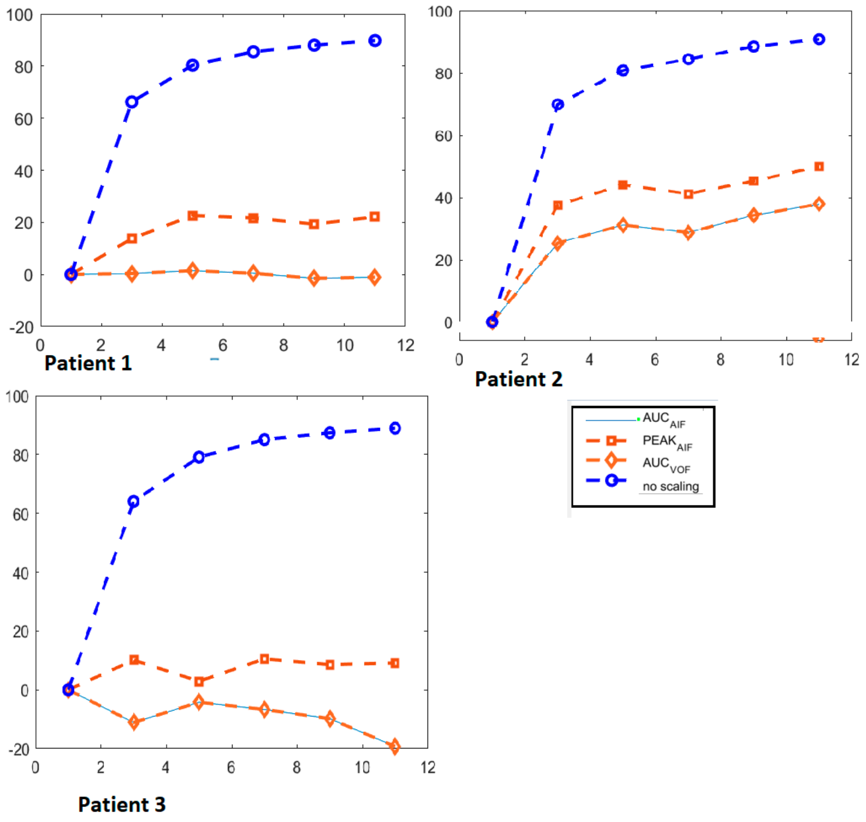

Figure S1: The association of increasing degree of PVE with deviation of CBF for three patients.

Figure S1 demonstrates the association of increasing degree of PVE with deviation of CBF for three individual patients i.e. the change in CBF (∆CBF) estimated from scaled AIF and Reference AIF (PVE present but minimal). Rescaling of the AIF using either of the three approach results in the reduced ∆CBF (%) values. We can see that this deviation or change in CBF after scaling by VOF(orange line) can either be nearly zero (patient 1), positive (patient 2) or negative (patient 3). From CBF deviation by VOF (orange line), we can observe that before scaling there was understimation of CBF values in patient 2 and overestimation of CBF values in patient 3. This implies that to generalize, we need a larger dataset to conclude if the scaling corrects the underestimation or overestimation in CBF values as this could vary patient to patient in a small cohort. However in the present cohort, utilizing scaling approach provides more reasonable absolute CBF/Tmax values, represented by the increased mean CBF/Tmax values and CBF/Tmax images.
